# Exosomal microRNAs (exomiRs): Small molecules with a big role in cancer

**DOI:** 10.1016/j.canlet.2018.02.002

**Published:** 2018-04-28

**Authors:** Rahul Bhome, Filippo Del Vecchio, Gui-Han Lee, Marc D. Bullock, John N. Primrose, A. Emre Sayan, Alex H. Mirnezami

**Affiliations:** a Cancer Sciences, University of Southampton, UK; b University Surgical Unit, University of Southampton, UK

**Keywords:** exomiR, exosome, microRNA, cancer, biomarker

## Abstract

Exosomes are secreted vesicles which can transmit molecular cargo between cells. Exosomal microRNAs (exomiRs) have drawn much attention in recent years because there is increasing evidence to suggest that loading of microRNAs into exosomes is not a random process. Preclinical studies have identified functional roles for exomiRs in influencing many hallmarks of cancer. Mechanisms underpinning their actions, such as exomiR receptors (“miRceptors”), are now becoming apparent. Even more exciting is the fact that exomiRs are highly suitable candidates for use as non-invasive biomarkers in an era of personalized cancer medicine.

## Highlights

• MicroRNAs packaged and secreted in exosomes are termed “exomiRs”.
• Sorting of miRNAs into exosomes is a selective process.
• Cancer and stroma-derived exomiRs influence many hallmarks of cancer.
• ExomiRs are highly promising cancer biomarkers.

## 1 Introduction

**Fig. 1:**
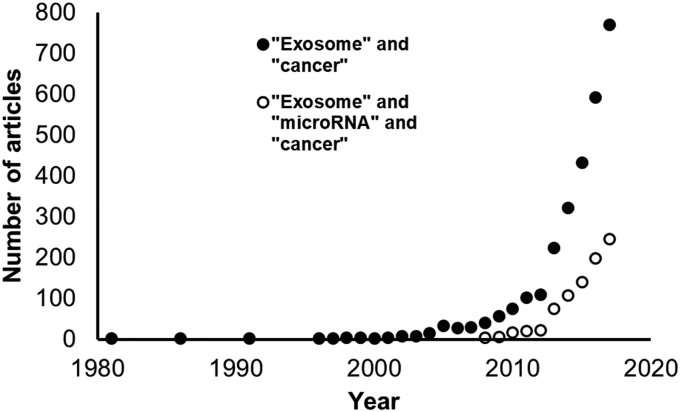
**Exosome-related research has risen exponentially.** Graphical representation of articles indexed by PubMed over time, containing the search terms **(a)** “exosome” and “cancer” and **(b)** “exosome” and “microRNA” and “cancer”.

There has been an exponential rise in exosome-related studies in the field of cancer biology (Fig. 1). This excitement was initially driven by exosomes as potential diagnostic and prognostic biomarkers [[1], [2], [3]] and has matured into an appreciation of the functional roles that exosomes play in processes such as pre-metastatic niche formation [4,5], metastatic organotropism [6] and therapy resistance [7]. MicroRNAs (miRNAs) are small non-coding RNAs referred to as master regulators of the genome [8], which are often dysregulated in cancer [[9], [10], [11], [12], [13]]. Their presence and intercellular transfer in exosomes has prompted deeper exploration of exosomal miRNAs (exomiRs), both as markers and signaling vehicles [[14], [15], [16], [17]]. We use the term “exomiRs” to describe miRNAs which are selectively packaged, secreted and transferred between cells in exosomes. This is an emerging field and far less is known in comparison to exosomes in general (Fig. 1). In this review we introduce exosome biology, discuss postulated mechanisms for miRNA loading into exosomes, highlight mechanistic roles of exomiRs in cancer progression and outline the biomarker potential of exomiRs in several common cancers.

## 2 Exosomes

### 2.1 Nomenclature

Exosomes are naturally occurring extracellular vesicles, ranging in size between 40 and 100 nm, with an endosomal origin [18]. However, the original definition by Trams and colleagues is much broader, encompassing all secreted vesicles with a biological function [19]. Technically speaking, exosomes are vesicles that sediment at 100,000 *g* [20]. Larger vesicles (greater than 100 nm) have been labeled microvesicles or microparticles, as vesicles which sediment at 10,000 *g* [[21], [22]]. Ectosomes, or shedding vesicles, are distinguished by their origin at and outward budding from the cell membrane [23]. These classes are not mutually exclusive, for example, microvesicles are ectosomes because they originate at the cell membrane [24]. Another level of complexity is added by naming vesicles according to their cargo, for example “oncosomes”, which contain oncogenic proteins [25]. Although the use of these different terms exists in the literature, the International Society for Extracellular Vesicles recommends use of the collective term “extracellular vesicles” (EVs) and strongly encourages researchers in the field to characterize their vesicles of interest by size, morphology and protein expression [26].

### 2.2 Biosynthesis and trafficking

Exosomes were first described in 1981 as a by-product of reticulocyte maturation [19]. In the ensuing decades, exosomes were shown to be secreted by a large variety of different cell types and we now believe that all cells produce exosomes [27,28].

**Fig. 2:**
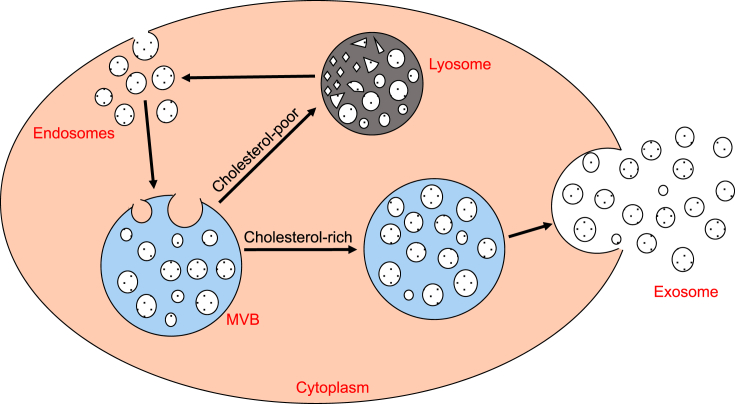
**Exosomes: extracellular vesicles with an endosomal origin.** Endosomes are packaged into MVBs, which are trafficked either to the cell membrane for secretion (cholesterol-rich) or to lysosomes (cholesterol-poor) for degradation. MVBs fuse with the cell membrane to release exosomes.

Exosomes are continuously released and recycled by cells. Through the process of endocytosis, exosomes re-enter cells, where they are called endosomes. Endosomes are packaged together in multivesicular bodies (MVBs). MVBs rich in cholesterol are trafficked to the cell membrane where they fuse and are released as exosomes, whilst those which are cholesterol deficient are recycled through lysosomes [29] (Fig. 2).

Intracellular transport systems involved in MVB packaging are thought to be highly conserved, resembling vacuole transport in yeast. Endosomal sorting complex responsible for transport (ESCRT) proteins such as ALIX and TSG101 are associated with this process [30]. ESCRT -0, I and II complexes recognize and sequester ubiquitinated membrane proteins at the endosomal membrane and ESCRT –III is responsible for cutting and inward budding [31]. However, combined knock down of ESCRT -0, -I, -II and -III still resulted in exosome production, suggesting that ESCRT-independent MVB packaging pathways exist [32].

Exosome release is thought to be dependent on intracellular calcium, Rab GTPases and SNARE proteins, although the precise coordination of events is unclear [[33], [34], [35]]. Rab11, Rab35 and Rab27a/b have been highlighted as key mediators of exosome release but it is still debatable whether they are redundant or whether any cell specificity exists [34,36,37]. Moreover, data suggest that SNAREs are important in the final interaction between MVB and cell membrane, based on our knowledge of lysosomal trafficking [35]. However, the specific complexes involved in this process are not thoroughly described.

Recipient cells take up exosomes by a number of mechanisms including endocytosis, micropinocytosis and phagocytosis. Endocytosis can be clathrin-mediated [38] or caveolin-dependent [39], and cholesterol-rich micro-domains in the cell membrane (lipid rafts) may facilitate this [40]. Micropinocytosis involves membrane invaginations which pinch off to draw extracellular content (e.g. fluid and exosomes) into the cytosol [41]. Phagocytosis of exosomes, which is more efficiently carried out by professional phagocytic cell types such as macrophages, is mostly PI3k-dependent [42]. Additionally, exosomes can directly bind to the recipient cell membrane and empty their contents [43].

Systemic injection of fluorescently labeled exosomes suggests that exosomes might be taken up non-specifically [44] but recent evidence suggests otherwise, for example, organotropic exosomes homing to specific sites by integrin-substrate interactions [6].

### 2.3 Exosome isolation and characterization

Exosomes are most commonly isolated from cell culture supernatant, blood or urine by differential ultracentrifugation, which involves sequential pelleting of contaminating cells (500 *g*), cellular debris (2000 *g*), apoptotic bodies and microparticles (10,000 *g*) and exosomes (100,000 *g*) [20]. Alternatively, a combination of filtration and ultracentrifugation can be used [45]. More recently, techniques such as size exclusion chromatography and Optiprep™ density gradient isolation techniques have emerged in an attempt to reduce contamination of exosome preparations with protein aggregates [46,47].

**Fig. 3:**
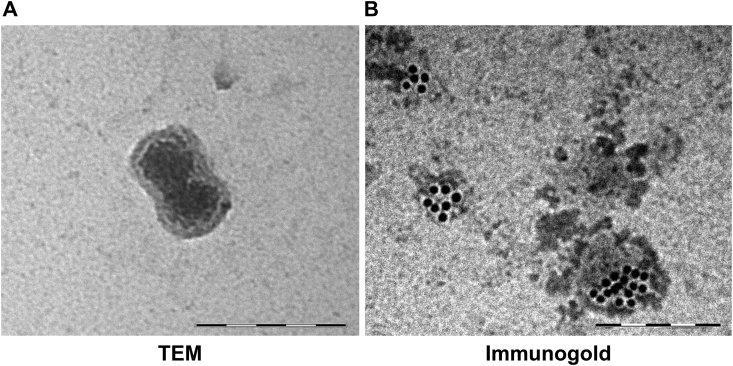
**Size, morphology and expression profile characterize exosomes. (A)** Transmission electron microscopy (TEM) of MRC5 fibroblast exosome (120 000x) demonstrating bi-layered structure. **(B)** Immunogold staining of primary mesenchymal stem cell exosomes with CD63. Scale bars in both panels represent 200 nm.

The International Society for EVs has published recommendations for EV characterization [26]. General characterization is typically done by protein expression; and at least three EV markers (e.g. ALIX, TSG101, CD63, CD81) should be enriched in the prep. Characterization of single vesicles by size is used to demonstrate the degree of heterogeneity in the sample. It is recommended that two techniques (e.g. electron microscopy and nanoparticle tracking analysis) are employed to show the uniformity of size distribution [48,49]. Fig. 3 shows exosome characterization by electron microscopy and immunogold staining. However, this is a field in flux and regular updates to the recommendations are expected [50]. To help standardize techniques, Van Deun and colleagues have recently developed the EV-TRACK knowledgebase [51]. This enables authors to deposit methodological parameters (e.g. source of EVs, rotor type, centrifugal force, protein markers) into a central repository, in exchange for an EV metric, which quantifies the robustness of their protocol.

### 2.4 Exosome-mediated RNA transfer

A major breakthrough in the field came in 2007, when it was shown for the first time that exosomes could transfer functional RNAs [14]. Valadi and colleagues isolated exosomes from MC/9 murine mast cells and co-cultured with HMC-1 human mast cells. Mouse-specific mRNAs and proteins were detectable in the human cells, suggesting that exosomes deliver mRNA which can be translated by the recipient cell machinery [14]. Interestingly, exosomes were found to contain large amounts of small RNAs, which were proven to be miRNAs. In the following years, transfer of miRNAs in exosomes has been demonstrated across multiple cell types [[52], [53], [54]].

## 3 Sorting of miRNAs into exosomes

### 3.1 Evidence for selectivity

**Fig. 4:**
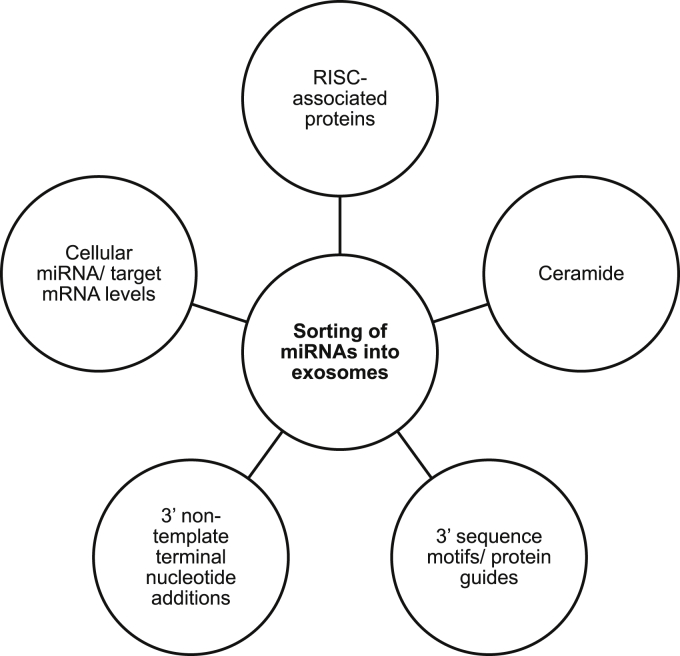
**ExomiR sorting is multi-faceted.** A schematic diagram to summarize postulated mechanisms for sorting of miRNAs into exosomes.

Goldie and colleagues showed that despite exosomes containing proportionally less small RNA than whole cells, the small RNA fraction was enriched in miRNAs [55]. This was shown by Guduric-Fuchs and colleagues to be specific to a subset of miRNAs, suggesting a selective loading mechanism [56]. Other studies have shown that exomiR profiles differ between cancer patients and healthy controls, suggesting that pathophysiological changes can modulate this mechanism [57]. In keeping with this, KRAS status of cancer cells can determine their exomiR profile [58]. Proposed mechanisms for exomiR sorting are outlined below and highlighted in Fig. 4.

### 3.2 RNA-induced silencing complex (RISC)

In 2009, two back-to-back articles highlighted the physical and functional association between miRNA-associated RISC proteins and MVBs [59,60]. Gibbings and colleagues separated conditioned medium from monocytes using Optiprep™ density gradient and showed that early fractions co-expressed MVB-associated markers and RISC proteins, specifically GW bodies (GW182, Ago2). This was confirmed using several combinations of immunofluorescent RISC-MVB markers [59]. Lee et al. knocked out *HSP4* in *Drosophila* with the effect of reducing MVB turnover [60]. MiRNA levels were significantly higher in Ago1 co-immunoprecipitates in these cells compared to wild type controls. These studies were the first to demonstrate the relationship between RISC and MVBs, raising the possibility that these proteins may be relevant in exomiR sorting.

### 3.3 Ceramide

Kosaka and colleagues were the first to show that exosomal miRNA content is regulated by ceramide [61]. In this study, the neutral sphingomyelinase-2 (nMase-2) inhibitor GW4869 was used to reduce ceramide biosynthesis in HEK293 cells. This resulted in a marked reduction in endogenous miR-16 and exogenous miR-146a in isolated exosomes. Although the nMase-2 inhibitor also reduced the quantity of cell-secreted exosomes, miRNA levels were still significantly lower after normalization. This was confirmed by knocking down and overexpressing nMase-2, which resulted in decreased and increased exomiR concentrations respectively. Importantly, genetic modulation had no effect on cellular miRNA levels. nMase-2 inhibition has since been used in several studies as a tool for reducing exomiR concentration [54,58]. It is worth mentioning that modulation of cell membrane constituents to alter exosome content should be viewed with caution. Exosomes are lipid-based vesicles; therefore, any step in their biogenesis, loading or uptake could be altered as a result of changes in cellular lipid homeostasis.

### 3.4 Sequence motifs and guide proteins

Using Jurkat cells, Villarroya-Beltri and colleagues discovered that 75% of exomiRs had a GGAG motif (extra seed sequence) at their 3′ end [62]. By applying site-directed mutagenesis to this motif in the predominantly exosomal miR-601, and transfecting this into Jurkat cells, they reduced its concentration in exosomes. Conversely, mutagenesis of the predominantly cellular miR-17, to include GAGG, led to an increased exosomal concentration. Exosome preparations from primary T-cells were then pulled down using streptavidin beads biotinylated with either an exomiR (miR-198) or a cellular miRNA (miR-17) and subjected to mass spectrometry to identify exomiR-linked proteins. Heterogeneous nuclear ribonucleoprotein (hnRNP) A2B1 was precipitated by the exomiR but not the cellular miRNA. Using electro-mobility shift assays, hnRNPA2B1 was shown to directly bind miR-198 but not mutant miR-198, or miR-17. Interestingly, the molecular weight of hnRNPA2B1 was found to be 10–12 kDa higher in exosomes compared to cells, and it was subsequently shown that this protein is sumoylated in exosomes.

Using a similar experimental approach on murine 3A hepatocytes, Santangelo et al. showed that miRNAs with a GGCU motif in their extra seed sequence bind to hnRNP-Q (also known as SYNCRIP) which guides them into exosomes [63]. Importantly, it was shown in this study that hnRNPA2B1 and hnRNP-Q bind selectively to miRNAs bearing respective GGAG or GGCU motifs, suggesting that there is sequence specific miRNA sorting into exosomes.

### 3.5 3′ end non-template terminal nucleotide additions (NTAs)

NTAs were previously found to be important in miRNA-RISC interactions [64]. Koppers-Lalic and co-workers investigated their role in exomiR sorting in B cells [65]. By RNA sequencing, they found that exomiRs were significantly more likely to be uridinylated at their 3′ end, whereas cellular miRNAs were more likely to be adenylated. The same findings were replicated in urinary exosomes of healthy individuals, suggesting that this phenomenon is not limited to B cells. Furthermore, this sorting mechanism was shown to apply to small cytoplasmic Y RNAs, and may be generalizable to other small RNAs.

### 3.6 Cellular levels of miRNAs and miRNA targets

De Palma's group transduced *Dicer*^*fl/fl*^ murine bone marrow-derived macrophages (BMDMs) with a Cre-expressing lentivirus to silence Dicer [66]. This disproportionately reduced exomiR levels compared to cellular miRNA levels. Conversely, overexpression of miR-511-3p, in immortalized BMDMs, led to a disproportionate increase in its exosomal levels. However, when artificial and naturally occurring (*Rock2*) target sequences complimentary to miR-511-3p were overexpressed in these cells, exosomal levels fell, suggesting that both cellular miRNA levels and miRNA targets determine exomiR sorting. To validate this, BMDMs were derived from *Lyz2.*Cre mice, which are deficient in lysozyme-2, a predicted target of the miRNA, miR-218-5p. As expected, their exosomes were shown to be more abundant in miR-218-5p compared to wild type BMDMs. Therefore, cellular availability of miRNAs has to be considered as a factor which determines the abundance of exomiRs.

## 4 Functional roles of exomiRs in cancer progression

### 4.1 Receptor-mediated exomiR signaling

On the understanding that viral small RNAs bind toll-like receptors (TLRs) in immune cells [67], Fabbri and colleagues discovered that exomiRs bind to TLRs in cancer cells to exert their effects [68]. Firstly, they co-cultured HEK293 cells overexpressing CD9-GFP with murine macrophages in which TLR-containing endosomes were labeled. CD9^+^ exosomes were internalized and were found to co-localize with TLRs. Next, TLR8-GFP was overexpressed in HEK293 cells and liposomal formulations of cy5-labeled miRNAs were applied to show co-localization of extracellular miRNAs to TLRs. Peritoneal macrophages from wild type and *TLR7*−/− mice were then exposed to liposomal miRNAs, showing that miRNAs stimulated cytokine production in wild type but not *TLR7* deficient cells, and thereby demonstrating a functional consequence of exomiR-TLR binding. Extrapolating this finding, it is plausible that exomiRs could also bind surface TLRs before being be internalized. This is one mechanism of exomiR signaling and others are likely to exist in parallel.

### 4.2 ExomiRs transfer phenotypic traits between cancer cells

Work from O'Driscoll's group previously showed that phenotypic traits such as invasiveness could be transmitted to recipient cells through exosome transfer [69]. Following on from this, Le and colleagues showed that exomiR transfer, specifically miR-200 family members, could influence metastatic capability in breast cancer cells [70]. Taking miR-200-rich exosomes from epithelial 4T1 cells and co-culturing with mesenchymal 4T07 cells, they were able to transfer miR-200 and downregulate ZEB2, reverting the 4T07 cells to an E-cadherin-expressing epithelial phenotype. When 4T07 cells were injected systemically with 4T1 exosomes, there was far greater lung colonization (metastases), suggesting that exosomal miR-200 transfer can drive mesenchymal-epithelial transition *in vivo*, allowing circulating tumor cells to seed at the secondary site.

### 4.3 Stroma-derived exomiRs influence cancer cells

Donnarumma and colleagues profiled exomiRs of patient-derived breast cancer-associated fibroblasts (CAFs) and normal fibroblasts (NOFs), and identified miR-21, miR-143 and miR-378 to be more abundant in CAF exosomes [71]. Using cy3-labeling they showed that these exomiRs could be transferred from CAFs to breast cancer cells in exosomes, resulting in enhanced mammosphere formation and expression of epithelial-mesenchymal transition (EMT) transcription factors. Exosomes from NOFs transfected with these exomiRs had the same effects on stemness and EMT. Supporting these data, Boelens and co-workers showed that fibroblast exosomes, containing non-coding RNAs, could induce RIG-I-STAT1 signaling in breast cancer cells, shifting the population to CD44^high^/CD24^low^ [7]. These cells had cancer stem cell attributes and were chemo- and radioresistant. We similarly profiled CAF and NOF exosomes from primary colonic tissue, proposing miR-329, miR-181a, miR-199b, miR-382, miR-215 and miR-21 as CAF-associated exomiRs in CRC [72]. Of these, miR-21 was the most abundant and enriched in exosomes compared to parental cells. Overexpression of miR-21 in normal fibroblasts co-injected with CRC cells led to significantly increased liver metastasis in an orthotopic mouse model. This suggests that exomiR transfer from CAFs has a meaningful function during cancer progression.

Beyond fibroblasts, Ono et al. attributed latency of metastatic breast cancer cells to exomiR transfer from bone marrow-derived mesenchymal stem cells (BM-MSCs) [73]. In this study, bone-tropic MDA231 cells were co-cultured with primary BM-MSC exosomes, leading to a decreased proportion of CD44^high^ cells. Having sorted the CD44^high^ cells, they showed that BM-MSC exosomes reduced proliferation and increased resistance to docetaxel. MiR-23b was found to be abundant in BM-MSC exosomes and its transfection into MDA231 cells recapitulated the observed effects on proliferation and stemness. These functional consequences were attributed to miR-23b repression of the cell cycle regulator MARCKS.

Challagundla and colleagues identified reciprocal exomiR transfer between neuroblastoma (NBL) cells and monocytes [54]. In this study, NBL cells were shown to deliver miR-21 to monocytes, stimulating M2 polarization, and through TLR8/NFκB activation, increasing monocyte secretion of exosomal miR-155. Monocyte exosomes were reciprocally taken up by NBL cells with resultant transfer of miR-155 and repression of the telomerase inhibitor, TERF1. As expected, xenografted subcutaneous tumors in cisplatin treated mice were significantly larger in the presence of injected liposomal miR-155.

## 5 ExomiRs as novel cancer biomarkers

### 5.1 The appeal of exomiR markers

For several years, miRNAs have been put forward as suitable diagnostic, prognostic and predictive biomarkers [[74], [75], [76], [77], [78]]. This is largely based on their ability to distinguish normal and malignant phenotypes, as well as different tumor types [9,79,80]. Equally, their stability in comparison to proteins and other nucleic acids, both in the circulation and in fixed tissues makes them particularly well-suited to sampling and analysis [81,82].

Circulating exomiRs may have added advantages as biomarkers over and above ‘free’ miRNAs. Firstly, exosome secretion from malignant tissue is greater than corresponding normal tissue, as evidenced by higher concentrations in biofluids such as plasma, urine and ascites [15,74,83,84]. Secondly, circulating exomiRs were shown to be representative of the parental tumor, in terms of miRNA profile [1,15,57]. Lastly, exosome-encapsulated miRNAs are highly protected from degradation, even in suboptimal storage conditions and in the presence of RNAse [85,86]. These factors may increase sensitivity of exomiR-based biomarkers. This is important because circulating tumor DNA (ctDNA) tests such as CancerSEEK, although demonstrating extremely high specificity, have been criticized for limited sensitivity (median 70%) [87]. A combination of ctDNA, protein and exomiR signatures may provide a solution to this problem in the future.

However, these potential advantages should be taken in context. The majority of circulating miRNAs are not exosomal but in fact bound to argonaute proteins [88]. Furthermore, stoichiometric analysis has revealed that in exosome preparations from plasma, there are over 100 exosomes for every abundant miRNA copy [89]. Despite this, the significantly increased load of circulating exosomes in the malignant state, coupled with the stability of miRNAs, has allowed the generation several putative exomiR markers. A selection of key studies pertaining to common cancers are summarized below.

### 5.2 Lung cancer

Rabinowits and co-workers highlighted the potential of exomiRs in their initial cohort of 27 stage I-IV lung adenocarcinoma patients and nine healthy controls [15]. Using a panel of 12 miRNAs previously associated with lung adenocarcinoma (miR-17-3p, miR-21, miR-106a, miR-146, miR-155, miR-191, miR-192, miR-203, miR-205, miR-210, miR-212 and miR-214), they showed that plasma exomiR profiles correlated with tumor-derived exomiR profiles, and all 12 exomiRs were more abundant in patients compared to controls.

More recently, Jin et al. tested the accuracy of plasma exomiRs in the diagnosis of stage I non-small cell lung cancer [16]. Using a combined NSCLC exomiR panel (let-7b, let-7e, miR-24 and miR-486) and individual panels for adenocarcinoma (miR-181b and miR-361b) and squamous cell carcinoma (miR-10b and miR-320b), they sampled the plasma of 60 symptomatic patients undergoing initial investigation. Receiver operating characteristic (ROC) curves produced area under the curve (AUC) values of 0.90 or greater for all panels.

### 5.3 Ovarian and breast cancer

Taylor's group was one of the first to demonstrate the utility of circulating exomiRs as diagnostic tools in ovarian cancer patients [74]. Using a previously validated signature of eight miRNAs (miR-21, miR-141, miR-200a, miR-200b, miR-200c, miR-203, miR-205 and miR-214), they showed that tumor miRNAs correlated with EpCAM-positive serum exomiRs, and that these could clearly distinguish ovarian papillary adenocarcinoma from benign ovarian disease in age-matched patients.

In breast cancer, Hannafon and colleagues profiled exomiRs from a normal mammary epithelial cell line (MCF10A) and multiple breast carcinoma lines (e.g. MCF7 and MDA231), and showed that miR-1246 was enriched in tumor-derived exosomes [90]. Using orthotopic patient-derived xenografts, they demonstrated that miR-1246 was more abundant in the plasma of implanted mice than controls, suggesting that tumor-derived exosomes contribute to the pool of circulating exosomes, which could be easily sampled. This was validated using plasma from patients with various subtypes of breast cancer compared to healthy controls. In terms of actually distinguishing breast cancer subtypes, Eichelser and co-workers showed that exosomal miR-373 in the serum was significantly increased in triple negative patients compared to those with luminal tumors or healthy controls [91]. Furthermore, transfection of plasmid encoding miR-373 into MCF7 cells led to reduced estrogen receptor expression, suggesting that this is a functional exomiR marker.

### 5.4 Prostate cancer

It was previously shown that miR-141 was elevated in serum of advanced prostate cancer patients [81]. Li et al. showed that miR-141 was enriched in serum exosomes compared to whole serum, and that levels were four fold higher in prostate cancer patients compared to those with benign prostatic hypertrophy or healthy controls [92]. Furthermore, in a prognostic capacity, this exomiR could distinguish localized from metastatic disease with greater than 80% sensitivity and specificity.

Huang et al. identified plasma exosomal miR-1290 and miR-375 to be associated with overall survival in castration-resistant prostate cancer, allowing them to develop a multivariate model including these exomiRs combined with prostate-specific antigen level and time to failure of hormonal therapy [93]. Similarly, Bryant and colleagues identified that plasma exomiRs could predict recurrence after radical prostatectomy [94]. In this cohort of 47 recurrent and 72 non-recurrent patients, miR-375 and miR-141 were increased in both plasma exosomes and microvesicles.

### 5.5 Colorectal cancer

Ogata-Kawata and colleagues identified 16 exomiRs which were more abundant in serum exosomes from CRC patients compared to healthy controls, and more abundant in conditioned medium from CRC cell lines compared to a normal colon line [1]. Using 29 paired pre- and post-resection samples, they selected and validated seven exomiRs (let-7a, miR-1229, miR-1246, miR-150, miR-21, miR-223, miR-23a), which were reduced following surgery. Each of these generated AUC values of 0.61 or more.

Using a similar approach, Matsumura et al. found that serum exomiRs which were more abundant in a recurrent case of CRC than a non-recurrent case, and miRNAs overexpressed in CRC tissue compared to normal colonic mucosa, converged on the miR-17-92 cluster [17]. In a validation cohort of 90 CRC patients and 12 healthy controls, miR-19a was more abundant in serum exosomes from CRC patients at all stages. In a separate cohort of over 200 CRC patients followed up for five years, circulating exosomal miR-19a was able to determine overall and disease-free survival.

## 6 Conclusion

The development of malignant cells, able to spread and populate distant microenvironments, is a complex and multi-step process, resulting from aberrant gene expression and cellular miscommunications, consequent to the accumulation of genetic and epigenetic abnormalities. The discovery of exosomes and their emerging and varied functions in biology and pathology undoubtedly represents one of the most exciting findings in the medical sciences in recent years. Unravelling their functions has exposed yet more complexity in the regulation of gene expression and cellular behaviour, and how normal mechanisms can become imbalanced in cancer. An ever increasing body of literature now attests to a link between these small packets of information, their non-coding RNA content and malignant disease, with their impact stretching across all described hallmarks of cancer.

Challenges in the field such as differing techniques for exosome isolation, tools to accurately quantify and characterize exosomal RNA, and demonstration of *in vivo* exomiR transfer, still remain [95]. Nonetheless, exomiRs are providing important clues and huge opportunities for diagnosis and prognostication. Early studies indicate that exomiR expression patterns can impact the biological behaviour of all cancers studied, and suggest that the clinical behaviour of many more tumors may be affected by the local, regional and systemic exosome and exomiR milieu. In the future, a greater dissection of the cellular and molecular pathways controlled by exosomes and their non-coding RNA cargo will undoubtedly provide exciting new insights into key neoplastic processes, and highlight promising areas for the development of novel therapeutic strategies such as vesicle-mediated gene therapy [96,97].

## Funding

This work was supported by the UK Medical Research Council [grant number MR/R002061/1].

## Conflicts of interest

The authors report no conflicts of interest.
